# Integrated pain care models and the importance of aligning stakeholder values

**DOI:** 10.1097/PR9.0000000000001160

**Published:** 2024-04-17

**Authors:** W. Michael Hooten, Miroslav Backonja, Kayode A. Williams, John A. Sturgeon, Jacob B. Gross, Sergey Borodianski, Victor Wang, Wen-Jan Tuan, Aleksandra E. Zgierska, Tobias Moeller-Bertram, Michael L. Kriegel

**Affiliations:** a Division of Pain Medicine, Department of Anesthesiology and Perioperative Medicine, Mayo Clinic, Rochester, MN, USA; b Division of Intramural Research, National Center for Complementary and Integrative Health, National Institutes of Health, Bethesda, MD, USA; c Division of Pain Medicine, Department of Anesthesiology and Critical Care Medicine, Johns Hopkins University School of Medicine, USA; d Johns Hopkins Carey Business School, Baltimore, MD, USA; e Department of Anesthesiology, University of Michigan Medical School, Ann Arbor, MI, USA; f Department of Anesthesiology and Pain Medicine, University of Washington, Seattle, WA, USA; g WellSpan Restorative Pain Program, York, PA, USA; h Boston PainCare, Waltham, MA, USA; i Department of Family and Community Medicine, Penn State College of Medicine, Hershey, PA, USA; j Department of Public Health Sciences, Penn State College of Medicine, Hershey, PA, USA; k Department of Anesthesiology and Perioperative Medicine, Penn State College of Medicine, Hershey, PA, USA; l Savas Health, Rancho Mirage, CA, USA; m Kriegel & Associates, Easley, SC, USA

## Abstract

Sustained widespread deployment of clinically and cost-effective models of integrated pain care could be bolstered by optimally aligning shared stakeholder values.

In a report from the Agency for Healthcare Research and Quality, 5% of the US population with complex chronic diseases account for 50% of total health care expenditures.^26^ Osteoarthritis and other nontraumatic joint disorders account for 44% of expenditures in this top 5% of patients. This is highly relevant to pain medicine because up to 68% of adults with high impact chronic pain have some form of arthritis^32^ that is incompletely responsive to conventual therapies.^28^ Thus, clinically effective and economically sustainable models of chronic pain care are urgently needed.

Integrated treatments for chronic pain are broadly based on the biopsychosocial model which posits pain as a reciprocating assimilation of physiological, psychological, social, and environmental factors.^2,9^ The biological underpinnings of the biopsychosocial model are supported by the pain matrix, which is recognized as a hierarchical multilevel neural network that processes painful stimuli.^15^ This processing spans the encoding of nociceptive stimuli and memory formation of pain experiences,^15^ which can be further modulated by immune and endocrine mechanisms.^33^ Although pain is an individual-specific experience, clinical symptoms represent a complex constellation of interrelated, multifaceted behaviors and environmental factors that are key drivers of pain-related impairments in physical, emotional, and social functioning.^21^ A broad array of integrated treatments has been described, but clinician access to these care models is limited, in part, by 2 key foundational barriers, which include (1) incomplete knowledge about the distinguishing characteristics of the most widely deployed models and (2) awareness of stakeholder alignment that is critical to achieving optimal value for all involved stakeholders. In this context, a stakeholder is generally defined as a person (eg, patient, clinician) or organization (eg, health care system, payer) with a vested interest in the treatment of chronic pain. The objectives of this commentary are two-fold. First, working definitions of multidisciplinary, interdisciplinary, and transdisciplinary pain care models will be provided from the perspective of 3 key stakeholder groups including the patient, clinician, and payer. Second, the engagement of each stakeholder in the care model will be conceptualized as a specific vector, defined as each stakeholder's pursuit of a desired value. The extent of vector alignment or convergence will be posited to yield the optimal goals of the care model for all engaged stakeholders.

Integrated pain care models generally extend the biomedical model of pain care where pain-targeted treatments are provided by individual clinicians. When other care modalities are indicated, patients are referred for specialty care. However, the lack of shared goals among stakeholders could impede the referral process. For example, patient preference could affect acceptance of the requested service, lack of clinician availability and coordination of care could delay scheduling, and limited or absent insurance benefits could avert the entire referral process even when patient acceptance and clinician availability are aligned.^20^ The limiting features of the biomedical model can be addressed by adopting more integrated models of care.

Multidisciplinary, interdisciplinary, and transdisciplinary care models can be conceptualized as anchors on a continuum of care characterized by the progressive integration of resources across disciplinary lines.^4,18,37^ The multidisciplinary model, which is the first anchor, is defined as 2 or more health care professionals from different disciplines sharing responsibility for decision making while working independently, in tandem or in parallel, to develop and implement care plans (Fig. 1, panel A).^12^ Multidisciplinary teams generally comprise pain medicine physicians, pain psychologists, primary care providers, nurses, pharmacists, physical therapists, and occupational therapists. Key components of this model include formation of collaborative action plans that fully encompass the exigencies of patients and effective team communication, usually in the form of clinic notes, focused on integrating the perspectives of each discipline. Individual practitioner vectors operate in a synchronous manner but continuing research suggests the lack of effective communication within the team, which partly relies on clinic notes, challenges optimal vector alignment which can adversely affect clinical outcomes.^5,30^ The clinical and cost-effectiveness of multidisciplinary pain care have been recognized for more than 2 decades,^11,14,19,41,42^ and more recent studies confirm these findings.^1,6,22,25^ However, widespread dissemination of multidisciplinary pain care has been curtailed,^7,36^ in part, by the model framework which is constructed without direct input from patients or payers. As a result, highly structured treatment protocols may not specifically address individual patient needs and, similarly, payers may be reluctant to reimburse for preselected services that have limited clinical relevance to some patient groups.^31,39^ Despite the critical shortcomings in the core architecture of the multidisciplinary care model, the synergy of partly aligning the payer vector was evident in a retrospective study where health care costs were significantly reduced in a group of adults receiving multidisciplinary care for a broad range of chronic pain diagnoses.^38^

**Figure 1.**
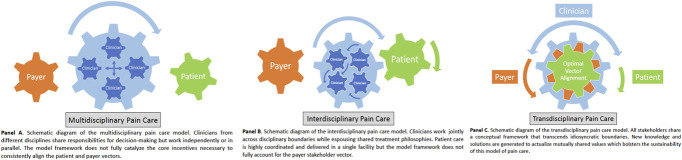
Multidisciplinary (panel A), interdisciplinary (panel B), and transdisciplinary (panel C) care models exist on a continuum of care characterized by the progressive integration of resources across disciplinary lines.

The interdisciplinary model, which is the second anchor on the integrated care continuum, is defined as 2 or more health care professionals working jointly across disciplinary boundaries while espousing shared treatment philosophies (Fig. 1, panel B). Although members of the health care team and treatment modalities are similar to the multidisciplinary model, an important distinguishing characteristic of the interdisciplinary model is the coordination and provision of patient care “under one roof at the same facility.”^16^ This ensures an environment of continual communication among team members which frequently includes care team meetings and discussion of treatment plans. Key elements of interdisciplinary care include teaching patients to use cognitive-behavioral techniques to modify maladaptive pain responses, graded exercise, and medication management to relieve the adverse effects of analgesic polypharmacy.^8,35^ Patients are generally expected to participate a highly structured treatment protocol spanning multiple weeks. The clinical outcomes,^3,13,27,43^ cost-effectiveness,^17^ and the process for accrediting interdisciplinary pain care facilities^24,27^ have been established.^3,13,17,27,43^ However, widespread deployment has been limited, in part, by the model framework which does not fully account for the payer vector.^8,29,36^ For example, in a prospective study of adults receiving interdisciplinary care for chronic pain, clinical outcomes were adversely affected by the lack of insurance benefits for physical therapy services.^34^

The optimal end point of the integrated pain care continuum is the transdisciplinary model which will be posited to provide a hitherto unrecognized opportunity to optimally align all stakeholder vectors (Fig. 1, panel C). This model is characterized by stakeholders sharing a conceptual framework that transcends idiosyncratic theories and concepts while simultaneously promoting the flexible exchange of discipline-specific roles to generate new knowledge and solutions for actualizing mutually shared values.^4,12,23^ When applied to delivering pain care, key stakeholders including patients and patient advocacy groups, clinicians, and payers engage from program inception to identify mutually shared values and then cooperatively design and implement effective treatment protocols. Similar to the interdisciplinary model, coordination and delivery of care occurs in a single facility, and patient schedules are flexible to accommodate patients' readiness to engage in subsequent stages of treatment. This flexibility also enhances the capacity to individualize treatment and promotes system resiliency by mitigating the impact of unanticipated events like missed appointments and provider absences. In addition, clinical communication and decision making are dynamic and not confined to scheduled team meetings. The recontextualization of patient engagement and treatment preferences, pursuit of evidence-based outcomes by clinicians, and incorporating measures of cost effectiveness and resource utilization by payers could further bolster the sustainability of this integrated care model. The potential synergistic effects of transdisciplinary care were reported in a single retrospective study involving 3296 patients with chronic pain.^40^ The stakeholder group that developed and deployed the treatment intervention was comprised of physicians, reconditioning specialists, complementary medicine and behavioral health experts, patients, and health insurance professionals (see Acknowledgements section in the study by Strigo et al.^40^ ) from a large managed care health plan. The 1-year transdisciplinary care intervention was organized into 3 phases (rescue, restore, and re-entry), and based on individual needs and preferences, patients were flexibly scheduled to receive care in 4 departments (medical, physical reconditioning, complementary care, and behavioral health) that worked in a truly integrated manner (see Supplemental Material 1 in the study by Strigo et al.^40^ for a program description). Evidence-based treatment plans were individualized based on patient expectations and choices.^10,40^ At 1-year follow-up, patients experienced significant improvements in pain interference, pain-related disability, pain catastrophizing, depressive symptoms, and anxiety symptom severity.^40^ Referrals to the transdisciplinary pain care program were initiated by the managed care health plan stakeholder and the costs were fully aligned with the payer's value of providing comprehensive pain care.

In summary, integrated pain care models are best conceptualized to exist on a continuum of care characterized by the progressive integration of knowledge and resources across disciplinary lines. Although the effectiveness of multidisciplinary and interdisciplinary models is widely recognized, widespread and sustained deployment has been limited, in part, by incomplete alignment of stakeholder vectors. By contrast, the transdisciplinary model is posited to provide an optimal framework for aligning key stakeholder vectors towards achieving mutually shared values at optimal levels. Strategic deployment of pain care centers of excellence based on the transdisciplinary model could accelerate widespread dissemination of this clinically effective and economically sustainable intervention for chronic pain.

## Disclosures

The authors have no conflicts of interest to declare.
